# Disulfiram inhibits liver fibrosis in rats by suppressing hepatic stellate cell activation and viability

**DOI:** 10.1186/s40360-022-00583-5

**Published:** 2022-07-22

**Authors:** Xiao-Mei Yang, Zheng Wu, Xiaoqi Wang, Yaoqi Zhou, Lei Zhu, Dongxue Li, Hui-Zhen Nie, Ya-Hui Wang, Jun Li, Xueyun Ma

**Affiliations:** 1 State Key Laboratory of Oncogenes and Related Genes, Shanghai Cancer Institute, Renji Hospital, Shanghai Jiao Tong University School of Medicine, Dongchuan Road, NO. 800, Shanghai, 200240 China; 2 Department of Radiation Oncology, Affiliated to School of Medicine, Renji Hospital, Shanghai Jiao Tong University, Shanghai, 200127 People’s Republic of China; 3 Institute of Biomedical Sciences, East China Normal University, Shanghai, 200241 People’s Republic of China

**Keywords:** Disulfiram, Liver fibrosis, Hepatic stellate cell, Co-culture

## Abstract

**Background:**

Liver fibrosis is a wound-healing response to chronic injury, featuring with excess accumulation of extracellular matrix secreted by the activated hepatic stellate cells (HSC). Disulfiram (DSF), also known as Antabuse, has been used for the treatment of alcohol addiction and substance abuse. Recently, overwhelming studies had revealed anti-cancer effects of DSF in multiple cancers, including liver cancer. But the actual effects of DSF on liver fibrosis and liver function remain unknown.

**Methods:**

In this study, we evaluated the effects of low-dose DSF in CCl4- and Bile Duct Ligation (BDL)—induced hepatic fibrosis rat models. Cell proliferation was detected by using the Cell-Light™ EdU Apollo®567 Cell Tracking Kit. Cell apoptosis was analyzed using a TdT-mediated dUTP nick end labeling (TUNEL) kit, viability was measured with Cell Counting Kit-8(CCK8). Relative mRNA expression of pro-fibrogenic was assessed using quantitative RT-PCR. The degree of liver fibrosis, activated HSCs, were separately evaluated through Sirius Red-staining, immunohistochemistry and immunofluorescence. Serum alanine aminotransferase (ALT) and asparagine aminotransferase (AST) activities were detected with ALT and AST detecting kits using an automated analyzer.

**Results:**

Liver fibrosis was distinctly attenuated while liver functions were moderately ameliorated in the DSF-treated group. Activation and proliferation of primary rat HSCs isolated from rat livers were significantly suppressed by low-dose DSF. DSF also inhibited the viability of in vitro cultured rat or human HSC cells dose-dependently but had no repressive role on human immortalized hepatocyte THLE-2 cells. Interestingly, upon DSF treatment, the viability of LX-2 cells co-cultured with THLE-2 was significantly inhibited, while that of THLE-2 co-cultured with LX-2 was increased. Further study indicated that HSCs apoptosis was increased in DSF/CCl4-treated liver samples. These data indicated that DSF has potent anti-fibrosis effects and protective effects toward hepatocytes and could possibly be repurposed as an anti-fibrosis drug in the clinic.

**Conclusions:**

DSF attenuated ECM remodeling through suppressing the transformation of quiet HSCs into proliferative, fibrogenic myofibroblasts in hepatic fibrosis rat models. DSF provides a novel approach for the treatment of liver fibrosis.

## Background

Hepatic fibrosis is deleterious and represents the common consequence of chronic liver diseases, which gives rise to a high risk of liver failure and hepatocellular carcinoma morbidity [1]. The persistent activation of the inflammatory response during chronic liver diseases induces activation of HSCs and results in excessive accumulation of scar tissues [2]. Over a long period, hepatic fibrosis has been defined as irreversible. However, more and more evidence has indicated that the direction of hepatic fibrosis might be turned over by various therapeutic strategies [3–5]. Unfortunately, the current treatment of hepatic fibrosis still lacks specificity and sensitivity.

Disulfiram (DSF) could inhibit the activity of aldehyde dehydrogenase (ALDH) to cause “disulfiram reaction” [6]. And due to the well-established pharmacokinetics, DSF has been recorded as an excellent safety profile, with rarely happened side effects [7, 8]. Recently, it has been reported that DSF has anti-cancer activities on several human cancers, including liver cancer [9–13], through its inhibition effects on ALDH. DSF has also been reported to combat diet-induced obesity in mice through the normalization of body weight [14]. The currently available oral version of DSF is quickly reduced to diethyldithiocarbamate (DDC) and degraded in the blood. One report using in vitro cultured HSC cell lines showed that DDC restrained collagen synthesis in HSCs via decreasing the level of reactive oxygen species (ROS) [15]. However, in vivo effects of DSF on hepatic fibrogenesis have not been elucidated.

In this study, we uncovered that low-dose DSF reduced the accumulation of collagen deposition in both CCl_4_- and BDL- induced hepatic fibrosis models. DSF alleviated liver fibrosis by suppressing the activation and viability of HSCs. Our results provided evidence that DSF might be a new candidate for clinical anti-fibrosis treatment.

## Materials and methods

### Cell culture and reagents

HSC-T6 cell was from the cell bank of the Chinese Academy of Sciences (Shanghai, China) and LX-2 was deposited in Shanghai Cancer Institute. THLE-2 was from American Type Culture Collection (ATCC, Manassas, VA) and cultured according to ATCC protocols. Disulfiram was purchased from Sigma (St. Louis, USA).

### Animal experiments

Animal experiments were performed as previously described [16]. Adult male Sprague–Dawley (SD) rats (about 200 g) were used for CCl_4_- or BDL-induced models. 16 rats were given intraperitoneal injections of 0.2 ml CCl_4_ diluted 1:1 in olive oil twice weekly and lasted for 8 weeks. One week after the first injection of CCl_4_, SD rats were randomly divided into two groups, one group received CCl4/vehicle and the other received CCl4/DSF intraperitoneally (n = 8 in each group). Similarly, rats were divided into two groups one week after BDL procedure and received DSF or vehicle treatment. DSF was dissolved with DMSO and injected twice a week (4 mg/kg body weight). Rats were euthanized by inhaling carbon dioxide in their cages and sacrificed 48 h after the last DSF injection. Rats were manipulated and housed according to protocols approved by the East China Normal University Animal Care Commission. All animals received human care according to the criteria outlined in the “Guide for the Care and Use of Laboratory Animals” prepared by the National Academy of Sciences and published by the National Institutes of Health.

### Isolation and culturing of rat HSCs

We used male SD rats (250 ~ 300 g) and a two-step collagenase/pronase E perfusion of rat livers followed by 18% Nycodenz two-layer discontinuous density-gradient centrifugation to isolate rHSCs. Briefly, rats were anesthetized and the vena cava and the vena porta were exposed. The vena porta was cut and inserted with a fine cannula which was placed properly and ligatured. The aorta abdominalis was cut after the first drops of blood out of the cannula, and then the liver was immediately in situ rinsed with sterilized Hank’s balanced salt solution (HBSS, without Ca2 + /Mg2 + , 37 °C) at a flow rate of 10 mL/min until liver turned grey. Subsequently, the liver was perfused with 90 mL of pronase E solution (Sigma, flow rate 10 mL/min, 37 °C), followed by 50 mL collagenase solution (Sigma, 37 °C) until the liver is greatly destroyed. The liver was further digested with 0.01 mg/ml DNase I solution containing 1% penicillin–streptomycin and separated into small pieces. Cell suspension was filtered through sterilized nylon meshes (100 μm) and subjected to centrifuge and resuspended in ice-cold HBSS (with Ca2 + /Mg2 +). Then Nycodenz (Sigma) density gradient centrifugation was performed (450 g, 4 °C) and isolated HSC cells were suspended in DMEM glutamax (Gibco) supplemented with 20% FBS and cultured on plastic dishes. Cells were replaced with fresh media every other day. Differential interference contrast images of cells were captured with a 20X objective lens at the indicated time points.

### Measurement of liver enzymes

Rat blood was collected in Eppendorf tubes and standing in 4℃overnight, followed by centrifugation at 3000 rpm/min for 20 min. The serum was collected and stored at -80℃. Serum ALT and AST activities were detected with ALT and AST detecting kits (Shen Suo You Fu Co. Ltd, Shanghai, China) using an automated analyzer.

### Quantitative RT-PCR

Total RNA was extracted using Trizol (Takara, China) and reverse-transcribed with the PrimeScript™ RT reagent Kit (Takara, China). Quantitative PCR was performed with SYBR Premix Ex Taq (TaKaRa, Japan). The primer sequences used were: ColI1a1, forward 5’CAGGCTGGTGTGATGGGATT3’, reverse 5’CCAAGGTCTCCAGGAACACC3’, TIMP1, forward 5’GACCACCTTATACCAGCGTT 3’, reverse 5’GTCACTCTCCAGTTTGCAAG3’, Acta2, forward 5’GGACGTACAACTGGTATTGTGC3’, reverse 5’CGGCAGTAGTCACGAAGGAAT3’, GAPDH, forward 5’GCTGAGTATGTCGTGGAGTCT3’, reverse 5’GGTTCACACCCATCACAAACA3’. Gene expression values were calculated based on the -△Ct method and normalized to GAPDH. Results were calculated as 2 ^−△Ct^ and normalized to the control group.

### Cell proliferation and viability assay

Cell proliferation was detected by measuring active DNA synthesis using the Cell-Light™ EdU Apollo®567 Cell Tracking Kit (RiboBio, Guangzhou, China). Isolated rHSCs were seeded in 48-well plates containing round coverslips at a density of 5000 cells/well and cultured for 2 days. Then the cells were pre-treated with vehicle or DSF for 48 h, followed by EdU incubation for another 48 h. Then the cells were fixed and visualized for EdU incorporation.

For cell viability assay, 100 μl of cell suspension (2000 cells/well) was inoculated in a 96-well plate. After incubation for 24 h, cells were treated with various concentrations of DSF for 48 h. Viability was detected with Cell Counting Kit-8 (CCK-8, Dojindo Molecular Technologies). Experiments were performed in triplicate and repeated twice.

For co-culture assay, 500 μl cell suspension of LX-2 (1*10^4^ cells/well) was inoculated in a 24-well transwell plate as LX-2 only, or co-cultured with 200 μl THLE-2 (5*10^4^ cells) cells inoculated in the upper chamber (pore size 0.4 μm). 12 h later, cells were replaced with 100 nM DSF or vehicle-containing medium and incubated for another 48 h. Then, the viabilities of LX-2 on the plate were detected using CCK-8. Reversely, to detect THLE-2 viability cocultured with LX-2, THLE-2 cells were inoculated below on the plate and LX-2 cells were on the upper chamber. Similarly, co-culture cells were treated with DSF or control and viabilities of THLE-2 were measured. Experiments were performed in triplicate and repeated twice.

### Sirius-red staining, immunohistochemistry and immunofluorescence

Preparation and staining of liver specimens were performed as previously reported [16]. Briefly, liver specimens were fixed in 10% formalin and embedded in paraffin. Then the sections were stained in 0.1% Sirius Red F3BA in a saturated picric acid solution. Randomly selected five fields from each section were photographed and analyzed. Red staining areas were quantified using NIH ImageJ software and expressed as a percentage of the total analyzed areas.

For immunohistochemical staining, primary antibodies for α-SMA (clone 1A4; Sigma-Aldrich), CD45 (PA5-87,427, Invitrogen) were incubated, followed by incubation of species-matched secondary antibody labelled with HRP (Thermo Scientific, USA) and detection with DAB substrate (Thermo Scientific). The nuclei were counterstained with hematoxylin.

For immunofluorescence, cells were cultured on BD Falcon™ 8-well Culture Slides, fixed and permeabilized, incubated with anti-alpha-SMA antibody and species-matched secondary antibody conjugated with Alexa Fluor-594. The nuclei were stained with DAPI (Sigma). Cell apoptosis was analyzed using a TdT-mediated dUTP nick end labeling (TUNEL) kit (R&D Systems, Inc.) according to the manufacturer’s instructions.

### Statistical analysis

Data were expressed as means ± SD. The student’s t-test was used for comparison between groups. Values of *P* < 0.05 were considered statistically significant.

## Results

### DSF alleviates liver fibrosis and liver injury in CCl_4_-induced rat hepatic fibrosis model

**Fig. 1:**
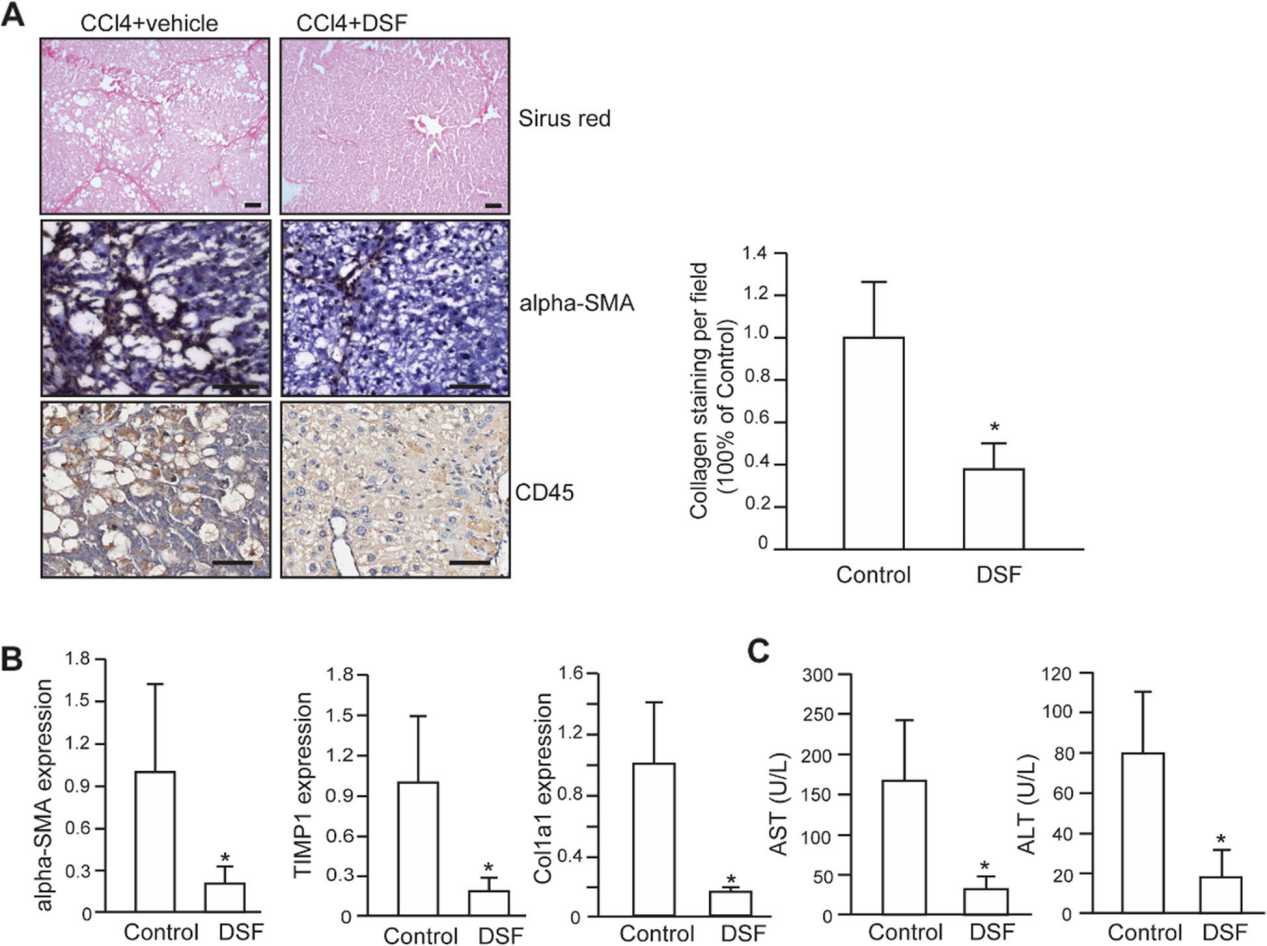
Effects of DSF on CCl_4_-induced fibrosis in rats. **A** Representative images of liver tissues stained with Sirius Red, α-SMA and CD45. Collagen deposition was indicated by the red strands. Statistical analysis of Sirus red-positive collagen per field was shown on the right panel. Scale bar, 50 μm. **B** Relative mRNA expression of pro-fibrogenic markers in liver, measured using qPCR. **C** Serum ALT and AST levels in DSF-treated and control group (*n* = 5). Data were expressed as means ± SD. *, *P* < 0.05

To directly investigate the effects of DSF during hepatic fibrogenesis, we established a CCl_4_-induced hepatic fibrosis model using adult male SD rats. One week after the first injection of CCl_4_, SD rats were randomly divided into two groups, one group received CCl4/vehicle and the other received CCl4 supplemented with low-dose DSF intraperitoneally (4 mg/kg body weight, *n* = 8 in each group). The definition of low-dose DSF was referred to and calculated according to previous reports [14, 17]. After 7 weeks, the rats were sacrificed and the livers were dissected and fixed. The degree of liver fibrosis was evaluated through quantification of Sirius Red-stained collagen areas in rat liver tissues. Compared with the control group, collagen deposition was significantly reduced in the DSF-treatment group (Fig. 1A).

Activation of HSCs has been recognized as a sign of hepatic fibrosis. Therefore, Actin Alpha 2, Smooth Muscle (ACTA2, also called α-SMA), a characteristic protein of HSC activation, was detected in rat liver specimens by immunohistochemical staining. The results showed that α-SMA staining was much weaker in DSF-treatment group compared to the control group (Fig. 1A). Given fibrogenesis is developed under close crosstalk between damaged hepatocytes, immune cells and HSCs, we detected the overall immune cells and found that CD45 positive cells were moderately decreased in DSF-treated group compared to the control group (Fig. 1A). Next, the levels of classical profibrogenic and hepatic function markers were examined. As shown in Fig. 1B, the expression levels of α-SMA, collagen type 1 α1 (Coll1a1), and TIMP metallopeptidase inhibitor 1 (TIMP1) were reduced in DSF-treated group. And notable reductions in ALT and AST levels were detected in rats treated with DSF (Fig. 1C). These data indicated that DSF has a protective effect on CCl_4_-induced hepatic fibrosis and liver injury.

### DSF reduces BDL-induced hepatic fibrogenesis

**Fig. 2:**
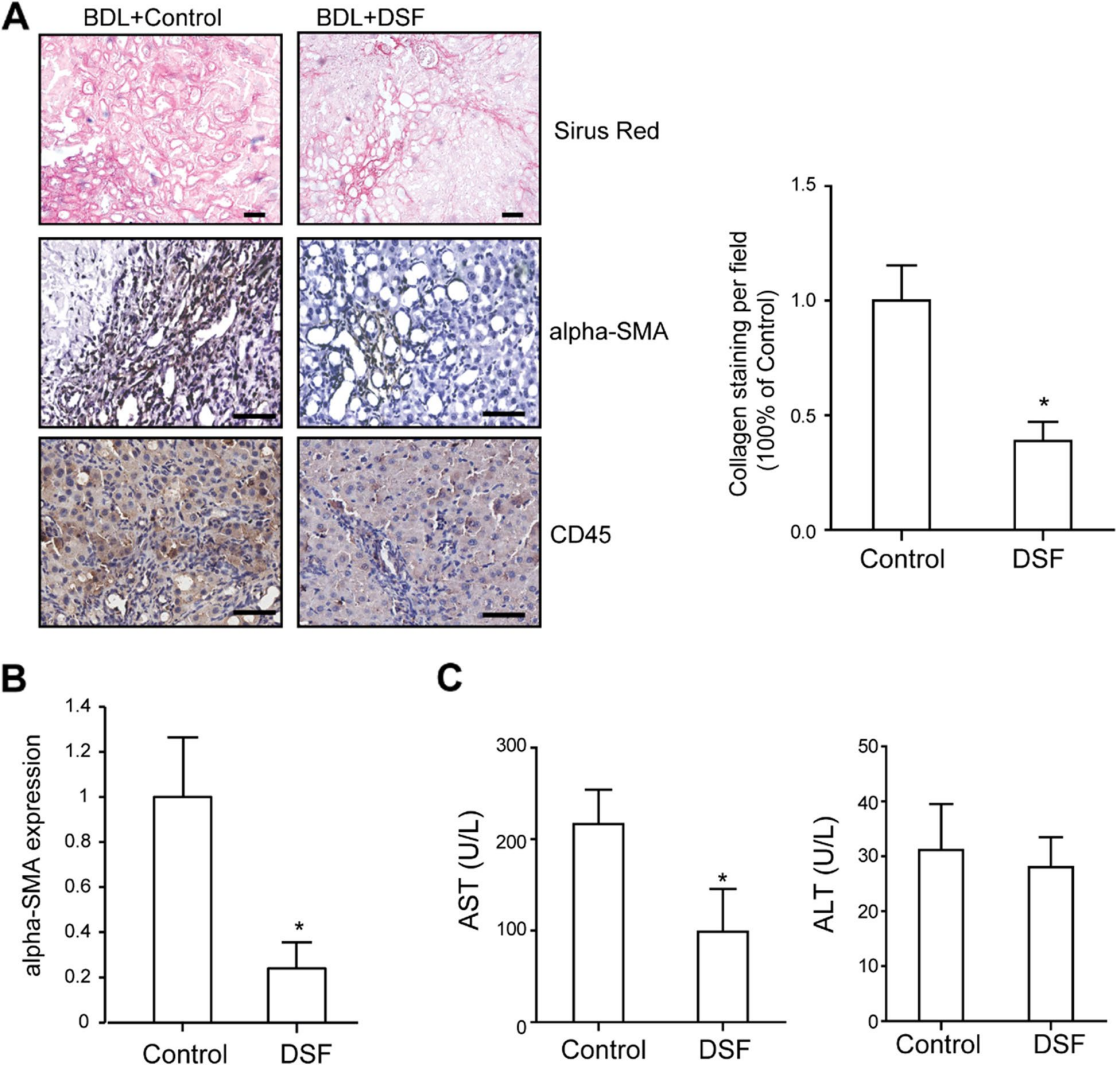
Effects of DSF on BDL-induced fibrosis in rats. **A** Representative images of liver tissues stained with Sirius Red, α-SMA and CD45. Statistical analysis of Sirus red-positive collagen per field was shown on the right panel. Scale bar, 50 μm. **B** Relative mRNA expression of α-SMA in liver, measured using qPCR. **C** Serum ALT and AST levels in DSF-treated and control group (*n* = 5). Data were shown as means ± SD. *, *P* < 0.05

Next, the BDL-induced liver fibrosis model was used to verify the effects of DSF on hepatic fibrosis. Consistent with the results in the CCl_4_-induced liver fibrosis model, BDL-induced liver fibrosis was also remarkably reduced via long-term and low-dose DSF treatment, as revealed by Sirius Red and α-SMA staining (Fig. 2A). Infiltration of CD45( +) overall immune cells were also moderately decreased by DSF treatment in this model (Fig. 2A). Quantitative PCR analysis further confirmed the reduced expression of α-SMA in DSF-treated group (Fig. 2B). Further, we found the serum level of AST but not ALT was decreased by DSF treatment (Fig. 2C). These findings confirmed that DSF attenuated rat hepatic fibrosis in vivo.

### DSF inhibits the activation and viability of primary rat HSCs in vitro

**Fig. 3:**
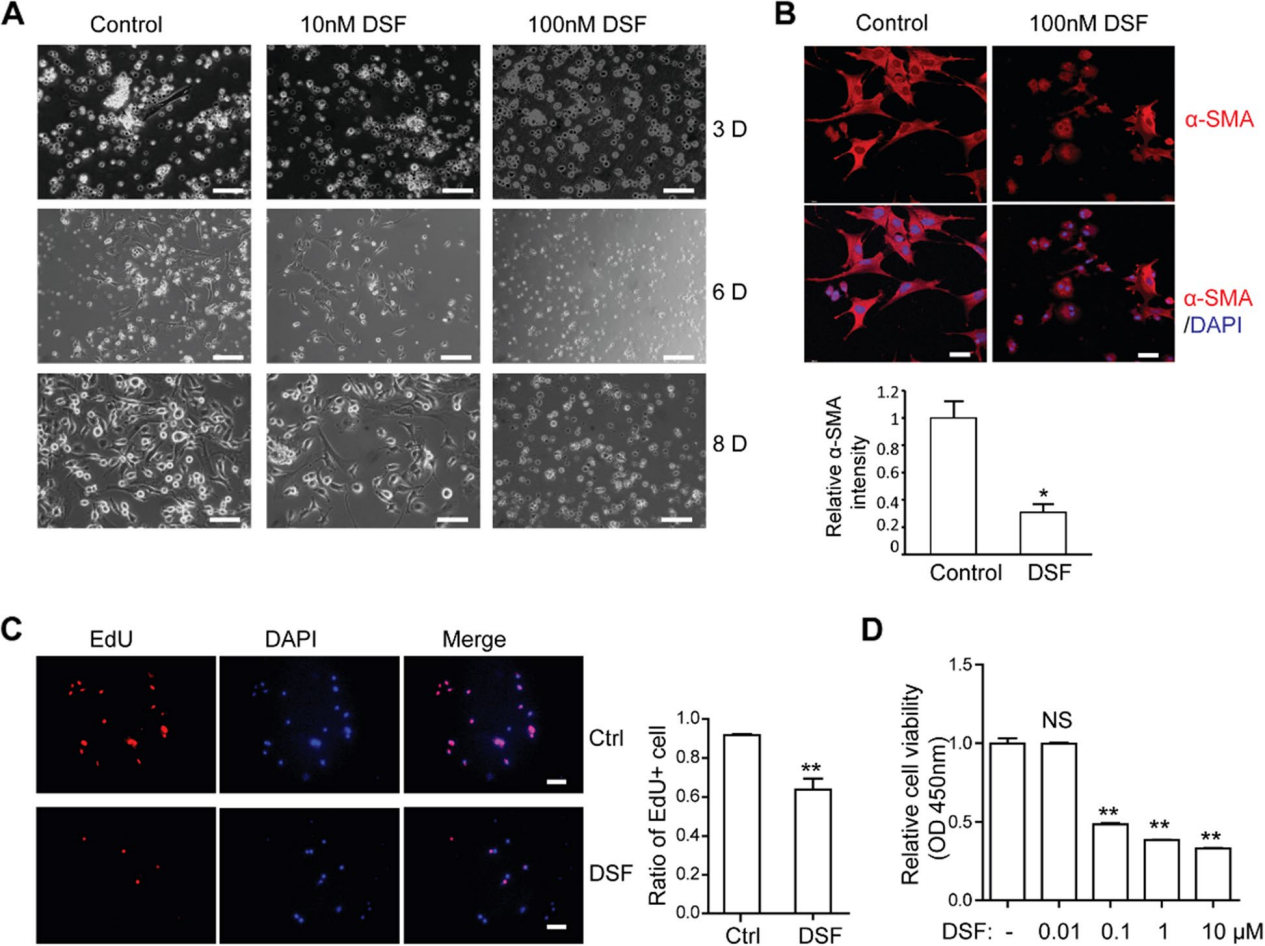
DSF inhibits the activation and viability of primary rHSCs in vitro. **A** Bright-field images of control and DSF-treated rHSCs at days 3, 6 and 8 of culture. Scale bar, 50 μm. **B** DSF-treated and control rHSCs were immunostained with α-SMA antibodies and DAPI. Quantified analysis of α-SMA intensities was shown below. *, *P* < 0.05 Scale bar, 25 μm. **C** EdU incorporation and quantification. EdU-positive cells were imaged on the left and quantified on the right panel. Scale bar, 50 μm. **D** Viabilities of rHSCs incubated with different concentrations of DSF **, *P* < 0.01

To investigate the effects of disulfiram on primary HSCs activation, we isolated rat HSCs (rHSCs) and cultured with DMEM/20%FBS on plastic dishes. Then, cells were changed with fresh complete medium containing vehicle control, or different concentrations of DSF every other day. To determine the effects of disulfiram on morphological changes associated with rHSCs activation, we monitored cell morphology at 3, 6 and 8 days after isolation. Cells treated with vehicles appeared to undergo the typical activation process of HSCs. These cells exhibited enlarged cellular bodies with increased cell numbers after 6 days of culture (Fig. 3A).

On the contrary, in DSF treatment groups, rHSCs activation was clearly suppressed with minimal morphological changes, especially in 100 nM DSF treated group (Fig. 3A). Moreover, α-SMA staining confirmed that rHSCs in the DSF treatment group were less activated than those in the control group, with weaker α-SMA and smaller cell sizes (Fig. 3B).

To investigate the effects of DSF on HSC proliferation, primary rHSCs were seeded in 48-well plates containing round coverslips and cultured for 2 days. Then the cells were pre-treated with vehicle control or 100 nM DSF for 48 h, followed by EdU incubation for another 48 h. As shown in Fig. 3C, EdU incorporation was distinctly decreased in the rHSCs treated with DSF. Primary rHSCs were also inoculated in 96-well plates and treated with different concentrations of DSF (0, 10 nM, 100 nM, 1 μM, 10 μM) for 24 h. The results showed that DSF inhibited primary HSCs viabilities dose-dependently, and 100 nM of DSF was sufficient to suppress primary HSCs viability (Fig. 3D). These data demonstrated that DSF inhibits the activation and proliferation of primary rHSCs dose-dependently.

### DSF inhibits the viability of HSCs but has minimal toxicity on hepatocytes

**Fig. 4:**
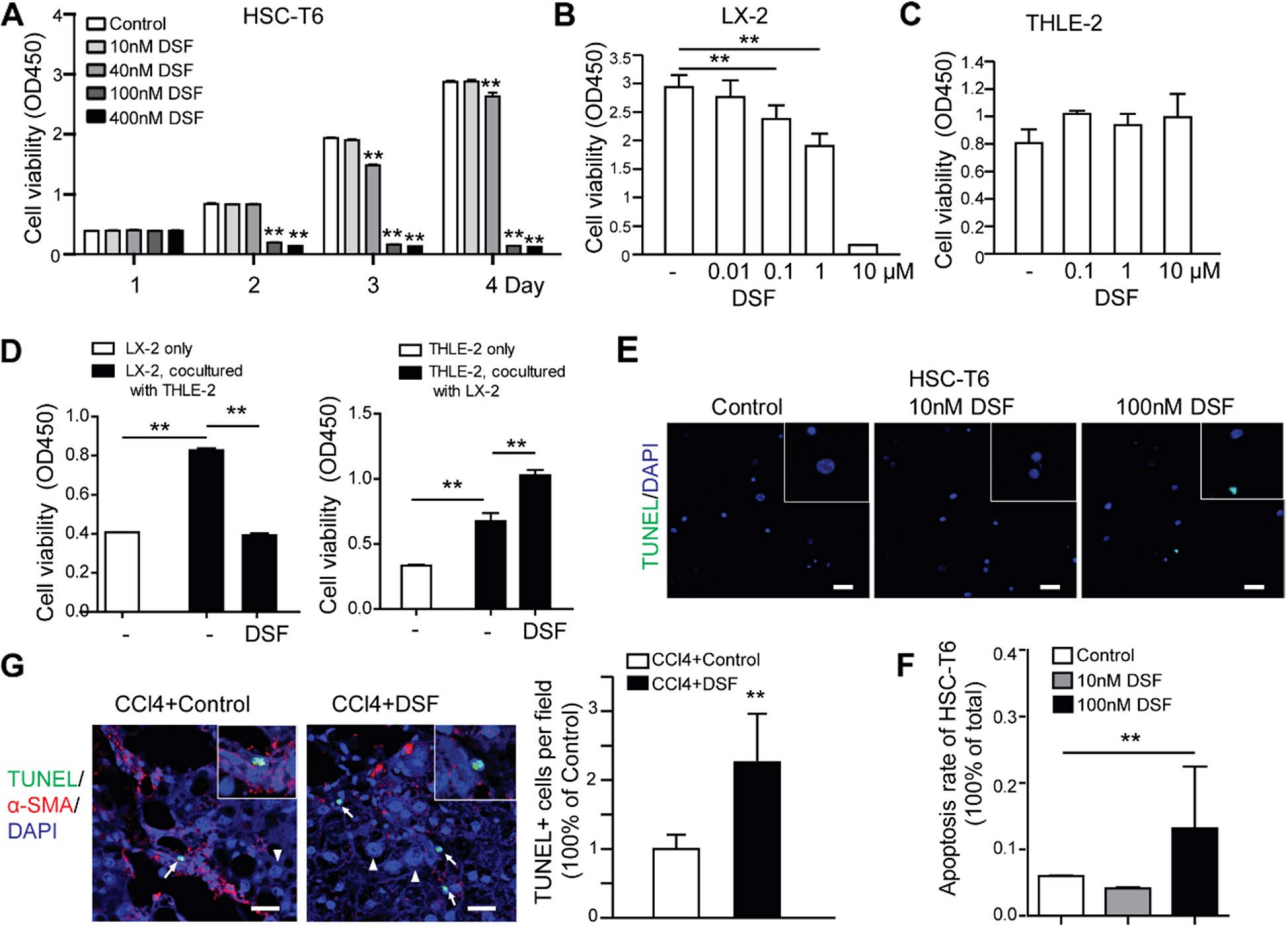
DSF inhibits the viability of HSCs but has minimal toxicity on hepatocytes. **A** Viability of HSC-6 T treated with increasing concentrations of DSF or control. **B, C** The viability of LX-2 (**B**) and THLE-2 (**C**) cells treated with DSF or control. **D** Cell viabilities of LX-2 only, or LX-2 co-cultured with THLE-2 treated with DSF or vehicle (left), and that of THLE-2 only, or THLE-2 co-cultured with LX-2 treated with DSF or vehicle (right). **E, F** HSC-T6 cell apoptosis was detected by TUNEL staining. Scale bar, 50 μm. The ratios of apoptotic cells were analyzed below. **G** Immunofluorescence staining of α-SMA (red) and TUNEL (green) in CCl4-induced fibrotic rat liver tissues. Close-up images were shown in the while rectangle. Scale bar, 25 μm. TUNEL-positive HSCs per field were analyzed on the right. White arrows indicated apoptotic HSCs and white arrowheads indicated hepatocytes. **, *P* < 0.01

Next, we investigated the effects of DSF on HSC cell lines. We found DSF also inhibited the viability of rat HSC cell line HSC-T6 and human HSC cell line LX-2 dose-dependently (Fig. 4A, B). Interestingly, DSF treatment did not suppress the viability of THLE-2, an immortalized hepatocyte cell line, even at a high concentration of 10 μM (Fig. 4C). To mimic the in vivo condition, LX-2 cells were inoculated in a 24-well transwell plate as LX-2 only, or co-cultured with THLE-2 cells (LX-2 down vs THLE-2 upper chamber, cell ratio, 1: 5). 12 h later, cells were replaced with 100 nM DSF or vehicle-containing medium and incubated for another 48 h. The result showed that the viability of LX-2 co-cultured with THLE-2 is evidently increased compared to LX-2 only, while it was significantly inhibited by DSF treatment (Fig. 4D, left).

Reversely, to answer whether DSF suppressed hepatocyte viability in a condition mimics in vivo liver tissue, THLE-2 only, or co-cultured with LX-2 was inoculated (THLE-2 down vs LX-2 upper, cell ratio, 5: 1) and treated with DSF as described above. The viability of THLE-2 co-cultured with LX-2 was increased compared with THLE-2 only, while it was surprisingly further increased by DSF treatment (Fig. 4D, right). These results indicate that DSF specifically inhibits the viability of HSCs in co-cultured status with a potential protective effect toward hepatocytes.

Next, using TUNEL staining, we investigated whether DSF promotes the apoptosis of activated HSCs. We found that the apoptosis ratio of HSC-T6 was significantly increased by 100 nM DSF in vitro (Fig. 4E, F). Moreover, CCl_4_-induced fibrotic rat liver tissues were subjected to co-immunofluorescence of α-SMA (red) and TUNEL (green) staining to simultaneously label HSCs and apoptotic cells. The results showed that the number of TUNEL-positive cells in α-SMA positive fibrosis regions was significantly higher in DSF-treated group than that in vehicle-treated group (Fig. 4G). These results indicated that DSF promotes apoptosis of active HSCs in vitro and in vivo.

## Discussion

The progress from chronic injury to liver fibrosis is accompanied by activation of hepatic myofibroblasts and ECM remodelling. With the increasing understanding of molecular mechanisms underlying liver fibrogenesis, novel strategies for blocking or reversing the fibrogenic progression are being investigated. Many antifibrotic agents have been proved to be effective in vitro or animal models, and several compounds showed safety profiles in phase 1 clinical studies [18], however, none has been approved in clinic practice. Another strategy to discover new antifibrotic drugs is to filtrate from existing agents used to cure other diseases [18, 19]. The benefit of this strategy is that many resources can be saved in analyzing the safety profile and pharmacokinetics of drugs.

DSF is an ALDH inhibitor, which is approved by the FDA for treating alcohol addiction [8]. DSF is able to suppress tumor growth through inhibition of proteasome activity [20, 21], abrogation of drug resistance [11, 22], or elimination of stem cell-like properties [23]. Recently, DSF is considered as an important drug for future HIV cure strategies, given its’ reversal effect on HIV latency [24]. By using CCl_4_- and BDL-induced rat hepatic fibrogenesis models, we found that DSF significantly attenuated liver fibrosis and hepatic injury. Subsequent assays indicated that DSF inhibited activation and proliferation of HSCs, and induced apoptosis of activated rat HSCs. Thus, DSF might be a good candidate to target HSC for suppressing liver fibrosis.

DSF has been recorded an excellent safety profile with rarely happened side effects [7, 8]. Liver failure was a much rare event for complicated reasons in humans receiving DSF, and it had not been observed in rats. To avoid possible toxicity, we used a much lower dose of DSF with long-term treatment in CCl4 and BDL models (4 mg/kg), compared to the doses in numerous reports in rats [25–27] and human trials[28–30]. Full-process administration of low-dose DSF may be more effective in inhibiting fibrosis, but higher dose of DSF administrated at a later stage might also work, which can be investigated in future.

The toxic concentration of DSF to hepatocytes is beyond micro-moles as revealed by in vitro studies in the literature [31, 32]. In this study, we applicated different concentrations of DSF and several sources of HSC to measure the effects on HSCs viabilities, and revealed the lowest concentration of DSF that effectively inhibited HSC viabilities. Excitingly, cytotoxicity was not observed in DSF-treated THLE-2 cells even at a concentration of 10 μM. And THLE-2 viability was even enhanced by DSF treatment while coculture with LX2, indicating that DSF specifically targets HSC with minimal toxicity on hepatocytes.

Coculture data also demonstrate that the crosstalk between hepatocytes and HSCs enhances their survival. These data are consistent with what we found in rat fibrosis models. Therefore, DSF has a distinctive anti-fibrosis effect through inhibiting HSCs activation and protecting hepatocytes. Nevertheless, the safety of DSF could be further confirmed by including a group of healthy control in the fibrosis models. And further investigations are needed to confirm the pro-apoptosis role of DSF by detecting the apoptosis signaling cascades and to reveal the underlying mechanisms of how DSF regulates HSC survival.

ALDH2 deficiency accentuated CCl_4_-induced hepatic fibrosis via ROS overproduction [33], while *ALDH2*^*−/−*^ mice were prone to get liver inflammation and fibrosis through malondialdehyde-acetaldehyde induced paracrine IL-6 activation in Kupffer cells [34]. Thus, as an ALDH inhibitor, DSF seems to aggravate fibrogenesis. However, our results showed that DSF suppressed hepatic fibrosis progression by inhibiting myofibroblast-like features of HSCs. Since ALDH is not the only target of DSF, we speculate that DSF might regulate HSCs activation through other mechanisms. It was reported that DSF administration resulted in the inhibition of CYP2E1 [35]. And several reports revealed that upregulated CYP2E1 in hepatocytes contributes to HSCs activation and fibrosis [36–38]. Thus, CYP2E1 might play a critical role in DSF-triggered antifibrotic progression, which needs further investigations.

Immune cells were recruited upon liver injury to mediate HSC activation during fibrogenesis[39]. Excessive inflammation was observed in CCl4- and BDL-induced fibrotic livers which were moderately reduced by DSF treatment. Thus, DSF might also directly suppress the infiltration of immune cells or reduce liver inflammation through inhibiting liver injury.

## Conclusions

As a treatment for alcohol addiction being used for over six decades, DSF has shown promising application prospects in other diseases including tumors. For the first time, we uncovered that DSF attenuated ECM remodeling through suppressing the transform of quiet HSCs into proliferative, fibrogenic myofibroblasts in hepatic fibrosis rat models. Our study indicated the antifibrotic effects of DSF, providing a novel approach for the treatment of liver fibrosis.

## Data Availability

All data used to support the finding of this study are included within the article.

## Abbreviations

DSF: Disulfiram
HSCs: Hepatic Stellate Cells
ECM: Extracellular Matrix
ALDH: Aldehyde Dehydrogenase
ROS: Reactive Oxygen Species
BDL: Bile Duct Ligation
ALT: Alanine Aminotransferase
AST: Asparagine Aminotransferase
SMA: Smooth Muscle Actin
